# *Haemaphysalis longicornis* and Carvacrol as Acaricide: Efficacy and Mechanism of Action

**DOI:** 10.3390/molecules30071518

**Published:** 2025-03-28

**Authors:** Na-Hyun Lee, Sangmin Lee, Namhyun Chung, Hoi-Seon Lee

**Affiliations:** 1 Department of Biotechnology, College of Life Sciences and Biotechnology, Korea University, Seoul 02841, Republic of Korea; nhlee0331@korea.ac.kr; 2 Department of Pharmacy Practice, College of Pharmacy, University of Illinois, Chicago, IL 60612, USA; slee7453@uic.edu; 3 Biomedical Research Team, HS Biotech for Medical Research, CH-4056 Basel, Switzerland

**Keywords:** *Haemaphysalis longicornis*, acaricidal activity, acetylcholinesterase, carvacrol, cytochrome P450 monooxygenases, detoxifying enzyme activity

## Abstract

Carvacrol derived from *Origanum vulgare* oil was evaluated for its acaricidal efficacy and mechanism of action against *Haemaphysalis longicornis*, a primary vector of severe fever with a thrombocytopenia syndrome. Essential oils extracted from *O. vulgare* leaves cultivated in Germany, Albania, and Iran were analyzed. Among them, the German oil exhibited the highest acaricidal potency due to its elevated carvacrol content (83.38%). Carvacrol was isolated and its identity was confirmed using GC/MS, NMR, and FT-IR analyses. Carvacrol demonstrated significant toxicity across all developmental stages of *H. longicornis*, with LC_50_ values of 3.47, 8.21, and 15.27 μg/cm^3^ for larvae, nymphs, and adults, respectively—representing 4.4-, 3.5-, and 3.2-fold higher potency compared to diethyltoluamide. The acaricidal mechanism of carvacrol involved multiple pathways: (i) inhibition of acetylcholinesterase activity by up to 85.4%, leading to neural disruption; (ii) suppression of cytochrome P450 monooxygenases (47.4% reduction), α-esterase (24.8% reduction), and β-esterase (28.6% reduction); and (iii) disruption of neural signaling pathways critical for survival. Chemical analysis confirmed carvacrol as the predominant active component in *O. vulgare* oil, with additional contributions from thymol and thymoquinone. Although its concentration in Iranian oil was lower, thymoquinone demonstrated the highest acaricidal potency (4.98 μg/cm^3^). Nevertheless, the abundance and superior efficacy of carvacrol establish it as the primary component contributing to the oil’s bioactivity. This study demonstrates that carvacrol is a promising eco-friendly alternative to synthetic acaricides for the control of *H. longicornis*. Its ability to inhibit acetylcholinesterase activity and suppress detoxifying enzymes suggests potential for overcoming resistance mechanisms associated with synthetic chemicals. Further studies should focus on optimizing the formulations and applying them in the field to improve efficacy.

## 1. Introduction

As industrial progress relentlessly exploits nature and the environment, new zoonotic diseases that affect both humans and animals are emerging. Zoonotic diseases are fatal conditions affecting both people and animals, resulting from environmental factors and caused by microorganisms, such as viruses, that can be transmitted between the two species [1,2]. The emergence and spread of zoonotic diseases have significantly impacted the world, as humans continue to harm nature and the environment, posing a significant threat today [1,2]. The World Organization for Animal Health states that 58% of infectious diseases in humans originate from animals, and more over 70% of infectious diseases impacting both humans and animals are zoonotic [2]. Recently, new zoonotic illnesses, such as severe fever with thrombocytopenia syndrome (SFTS), monkeypox, and Ebola, have rapidly and significantly spread among humans and animals in Africa and Asia [3].

The Asian long-horned tick, *Haemaphysalis longicornis* Neumann (Ixodida: Ixodidae), 1901, is a significant vector for the SFTS virus and has been increasingly recognized for its role in transmitting various pathogens. *H. longicornis*, commonly found in Korea, China, Japan, and Australia, is recognized as the primary vector of the SFTS virus among zoonotic infectious diseases [3]. Patients infected with the SFTS virus exhibit the most severe symptoms, including high fever, leukopenia, and thrombocytopenia [3]. In Korea, the peak incidence of SFTS virus infections in humans and animals attributed to *H. longicornis* occurs from July to November, with a human case fatality rate of 18–30% associated with the SFTS virus [4]. Unfortunately, there is now no treatment or vaccine for the symptoms of SFTS caused by the vector *H. longicornis*, and we are actively investigating methods to manage *H. longicornis*, the pathogen responsible for this infectious disease. Recently, due to the significant changes in the natural environment caused by substantial climate change, the incidence of *H. longicornis* infected with SFTS virus is rapidly and severely increasing. The concerning increase in SFTS virus infections suggests that it may pose a significant global threat in the future. Therefore, preventing *H. longicornis* from accessing humans and animals has lately become essential in the field of zoonotic diseases.

Until recently, the control of *H. longicornis* was mostly achieved by the use of synthetic acaricides. The rampant and uncontrolled use of synthetic acaricides has led to significant environmental degradation, resistance, and non-target toxicity [5]. In light of these issues, many scientists are actively developing environmentally sustainable, natural acaricides to protect humans and animals from *H. longicornis* [5]. While other studies have examined the acaricidal characteristics of several plant-derived chemicals, no research has explicitly assessed the efficiency of carvacrol against *H. longicornis*. This study conducts a thorough assessment of carvacrol, a principal constituent of *O. vulgare* oil, as an acaricidal agent against *H. longicornis* nymphs, examining its potential mechanism of action via enzymatic inhibition, thus offering new perspectives on sustainable tick control methods.

This study conducted the following research findings: (i) to evaluate the acaricidal efficacy of essential oils extracted from *O. vulgare* leaves cultivated in Germany, Albania, and Iran against *H. longicornis*; (ii) to analyze the components of these essential oils and assess the acaricidal activities of individual constituents against *H. longicornis* nymphs; (iii) to isolate and identify the active constituent of *O. vulgare* oils; and (iv) to investigate enzymatic activities against acetylcholinesterase (AChE) and detoxifying enzymes in *H. longicornis* nymphs.

## 2. Results and Discussion

### 2.1. Acaricidal Activities of O. vulgare Oils

The yield of essential oils extracted from *O. vulgare* leaves using steam distillation was determined to be 0.21%, 0.27%, and 0.24% for leaves grown in Germany, Albania, and Iran, respectively. The acaricidal efficacy of essential oils was evaluated against *H. longicornis* larvae, nymphs, and adults (Table 1). To assess their effectiveness, the results were compared with the acaricidal activity of diethyltoluamide, a commonly used synthetic acaricide serving as a positive control. The essential oil derived from *O. vulgare* leaves in Germany displayed acaricidal properties, as indicated by the 50% lethal concentration (LC_50_) from the immersion assay, with LC_50_ values of 3.53, 12.32, and 18.14 μg/cm^3^, demonstrating efficacy approximately 4.4, 2.3, and 2.7 times superior to diethyltoluamide, which has LC_50_ values of 15.34, 28.61, and 49.21 μg/cm^3^ against *H. longicornis* larvae, nymphs, and adults, respectively (Table 1). The Albanian oil (LC_50_, 4.49, 13.86, and 21.02 μg/cm^3^) exhibited acaricidal activity approximately 3.4, 2.1, and 2.3 times more effective than diethyltoluamide against *H. longicornis* larvae, nymphs, and adults. The essential oil extracted from the leaves of *O. vulgare* in Iran exhibited acaricidal activity with an LC_50_ of 5.27, 15.02, and 25.29 μg/cm^3^, demonstrating efficacy about 2.9, 1.9, and 2.0 times superior than diethyltoluamide against the same mites. No mortality was observed in the negative control group regarding *H. longicornis* larvae, nymphs, and adults. The results indicate that the acaricidal efficacy of German *O. vulgare* oil surpasses that of Albanian and Iranian oils across all assessed mites. A substantial body of research demonstrates that geographical and environmental factors significantly influence the quality of plant oils. Previous studies have demonstrated that variations in oil quality, efficacy, and chemical composition substantially affect acaricidal efficiency, as seen by the changes in essential components influencing *Dermatophagoides* spp. and *T. putrescentiae* [6].

### 2.2. Chemical Composition of O. vulgare Oils

To further elucidate the major components of essential oil extracted from *O. vulgare* leaves cultivated in Germany, Albania, and Iran and to determine which major components have acaricidal efficacy against *H. longicornis* nymphs, the components of the aforementioned oils were identified via GC/MS analysis. The components investigated by the area percentage, retention index, and retention time in the GC/MS analysis are displayed in Table 2. The chemical classification of *O. vulgare* oils includes monoterpene hydrocarbons (α-terpinene, cymene, γ-terpinene), monoterpene alcohols (borneol, linalool), phenols (thymol, carvacrol), quinones (thymoquinone), and sesquiterpenes (caryophyllene). Ten components were found in the essential oils from Germany, Albania, and Iran, accounting for 99.37, 99.51, and 97.38% of the total oil content, respectively. The principal constituents of the German oil were determined to be carvacrol (83.38%), followed by cymene (4.94%), thymol (4.72%), γ-terpinene (2.77%), linalool (2.36%), borneol (0.49%), caryophyllene (0.41%), and α-terpinene (0.30%). The principal constituents of the Albanian oil were discovered as carvacrol (79.66%), followed by thymol (5.74%), cymene (5.59%), γ-terpinene (3.33%), linalool (2.98%), caryophyllene (1.75%), and α-terpinene (0.46%). The principal constituents of the Iranian oil were identified as carvacrol (63.86%), followed by thymol (8.68%), cymene (6.34%), caryophyllene (5.52%), γ-terpinene (5.36%), linalool (4.98%), α-terpinene (2.41%), and thymoquinone (0.23%). The individual constituents of the essential oils were assessed against *H. longicornis* nymphs (Table 3), yielding LC_50_ values of 10.37, 8.21, and 4.98 μg/cm^3^ for thymol, carvacrol, and thymoquinone, respectively. The LC_50_ of thymol, carvacrol, and thymoquinone exhibited acaricidal potency against *H. longicornis* nymphs that was 2.8, 3.5, and 5.8 times superior to diethyltoluamide, respectively. The subsequent analysis revealed that benzene, α-terpinene, cymene, γ-terpinene, borneol, linalool, and caryophyllene demonstrated no acaricidal efficacy against *H. longicornis* nymphs. Thymol and thymoquinone demonstrated acaricidal properties; nevertheless, their quantities were rather low, indicating a limited impact on the oil’s overall toxicity profile.

This study explores the chemical composition of *O. vulgare* oil from three geographical regions—Germany, Albania, and Iran—and evaluates its acaricidal efficacy against *H. longicornis* nymphs. GC/MS analysis revealed that carvacrol was the predominant component across all three oils, with the highest concentration observed in German oil (83.38%), followed by Albanian oil (79.66%) and Iranian oil (63.86%). Despite the differences in secondary constituents among the samples, carvacrol consistently comprised the largest proportion of the oil’s composition, highlighting its critical role in bioactivity. Among the ten identified components, only carvacrol, thymol, and thymoquinone exhibited acaricidal activity. Thymoquinone demonstrated the highest toxicity with an LC_50_ of 4.98 μg/cm^3^, making it 5.8 times more potent than diethyltoluamide and 3.7 times more potent than permethrin. Carvacrol (LC_50_, 8.21 μg/cm^3^) and thymol (LC_50_, 10.37 μg/cm^3^) also showed significant acaricidal effects but were less toxic than thymoquinone. These results align with prior studies indicating that phenolic compounds such as carvacrol and thymol disrupt neural signaling in arthropods by interfering with octopaminergic and GABAergic pathways, leading to neurotoxic effects [7]. Thymoquinone was exclusively detected in Iranian oil, while borneol was unique to German oil. However, the low concentration of thymoquinone in Iranian oil (0.23%) suggests it contributes minimally to the overall acaricidal activity of the oil. Similarly, thymol was present in low concentrations across all three oils despite its moderate toxicity. The strong acaricidal efficacy of *O. vulgare* oil is primarily attributed to its high carvacrol content, which is known to disrupt membrane permeability and inhibit enzymatic functions essential for arthropod survival [8,9]. This study underscores how the geographical and environmental factors influence the chemical composition of essential oils, resulting in affecting their bioactivity. As *H. longicornis* becomes increasingly resistant to synthetic acaricides and environmental toxicity becomes a concern, carvacrol-rich *O. vulgare* oil emerges as a promising natural alternative for tick control. Selecting carvacrol-dominant oils is crucial for maximizing acaricidal effectiveness considering regional variations that may influence their overall toxicity profiles. These findings highlight the potential of essential oils as sustainable solutions for pest management in light of the increasing resistance to synthetic chemicals.

### 2.3. Isolation and Identification of the Active Component

The acaricidal activity of *O. vulgare* oil against *H. longicornis* nymphs led to the isolation of its active compound, OV43342. This compound was purified using silica gel chromatography and recycling prep HPLC. Its structure was elucidated through spectroscopic techniques. The EI/MS spectrum was analyzed, and the peak indicating the molecular weight was found to be 150 (40), which corresponds to the molecular formula C_10_H_14_O. MS m/e (rel. int.): 150 (40), 135 (100), 127 (2), 117 (8), 107 (9), 91 (12), 84 (1), 77 (8), 65 (3), 58 (1), 51 (2), 39 (3), and 27 (2). The ^13^C NMR spectrum of purified carvacrol exhibited 10 distinct peaks, confirming the presence of diverse carbon environments. The downfield signals at δ 153.5 and δ 148.4 were assigned to sp^2^-hybridized carbons within the hydroxyl-substituted aromatic system, indicative of the electron-donating effect of the hydroxyl (-OH) group. Additional signals at δ 120.6, δ 130.8, δ 118.8, and δ 112.9 correspond to sp^2^-hybridized aromatic carbons, confirming substitution patterns within the benzene ring. The presence of a benzylic methylene (-CH_2_-) group was supported by a characteristic signal at δ 33.6, which is deshielded due to its proximity to the electron-rich aromatic system. Furthermore, three methyl (-CH_3_) carbons were identified at δ 24.1, δ 23.9, and δ 15.3, indicating alkyl branching within the aliphatic framework. The ^1^H NMR spectrum provided further insight into the proton environments of carvacrol. Aromatic proton signals appeared as doublets at δ 6.944–6.966 (J = 13.2 Hz) and δ 6.633–6.647 (J = 8.4 Hz), corresponding to meta- and ortho-coupled protons, respectively. Additionally, a singlet at δ 6.599 was attributed to an isolated aromatic proton, likely positioned between the hydroxyl and isopropyl (-CH(CH_3_)_2_) substituents. In the aliphatic region, a multiplet at δ 2.705–2.751 was assigned to benzylic CH_2_ protons, indicative of a methylene group adjacent to the aromatic ring. A singlet at δ 2.216 confirmed the presence of a methyl (-CH_3_) group directly attached to the benzene ring. Additionally, a singlet at δ 1.119–1.130 (J = 6.6 Hz) was assigned to the methyl groups of the isopropyl (-CH(CH_3_)_2_) moiety, further supporting the substitution pattern. The combination of ^13^C and ^1^H NMR spectral data confirms the structural features of carvacrol, including the hydroxyl-functionalized benzene ring, benzylic methylene, and alkyl branching. These findings are consistent with previously reported spectral data for carvacrol, reinforcing the molecular identity and substitution patterns of the compound. FT-IR supported these findings by confirming functional groups characteristic of phenolic compounds. A broad absorption band between 3200 and 3600 cm^−1^ indicated the presence of a hydroxyl (-OH) group with strong hydrogen bonding interactions. Aliphatic C-H stretching vibrations were observed between 2950 and 2850 cm^−1^, while aromatic C=C stretching vibrations appeared in the range of 1600–1450 cm^−1^. A distinct C-O stretching vibration was detected between 1200 and 1000 cm^−1^, consistent with a phenol functional group. Out-of-plane C-H bending vibrations in the range of 900–700 cm^−1^ confirmed an ortho-substituted benzene system. DEPT NMR distinguished CH, CH_2_, and CH_3_ groups within OV43342, supporting assignments made from ^13^C NMR data. HMQC NMR established direct ^1^H-^13^C correlations between protons and their respective carbons, providing insights into connectivity between aromatic and aliphatic regions of the molecule. COSY NMR revealed scalar couplings between adjacent protons in both aromatic and aliphatic regions, further clarifying structural connectivity. Through these combined spectral analyses, OV43342 was identified as carvacrol—a phenolic monoterpenoid with hydroxyl-substituted benzene rings and aliphatic methyl/methylene groups consistent with its molecular framework. Carvacrol’s structural features include a hydroxyl group on an aromatic ring and branching aliphatic substitutions. Mass spectrometry confirmed carvacrol’s identity based on its molecular ion peak and fragmentation pattern matching the previously reported data for this compound. Carvacrol is known for its bioactivity; prior studies reported its acute ingestion toxicity against *Drosophila melanogaster* nymphs with an IC_50_ of 0.175 mM [10]. The spectral data obtained in this study align closely with these findings. In conclusion, carvacrol was successfully isolated from German oil as the active acaricidal constituent against *H. longicornis*.

### 2.4. Acaricidal Activities of Active Components Against H. longicornis

Considering the acaricidal properties of carvacrol and thymol derived from *O. vulgare* oil, as well as the concentration of acaricidal constituents affecting efficacy, two compounds were selected for investigation to assess their acaricidal activity against *H. longicornis* larvae, nymphs, and adults. Their acaricidal properties were compared with the LC_50_ and LC_90_ values of diethyltoluamide and permethrin, which operate as positive controls against *H. longicornis* larvae, nymphs, and adults (Table 4). According to the LC_50_ values obtained from the immersion assay, carvacrol exhibited approximately 4.4-, 3.5-, and 3.2-folds greater efficacy than diethyltoluamide against *H. longicornis* larvae, nymphs, and adults, respectively. According to the LC_50_ values for *H. longicornis* larvae, carvacrol (LC_50_, 3.47 μg/cm^3^) exhibited the most effective acaricidal activity, surpassing diethyltoluamide (LC_50_, 15.31 μg/cm^3^), followed by thymol (LC_50_, 5.26 μg/cm^3^) and permethrin (LC_50_, 10.24 μg/cm^3^). When evaluating the LC_50_ values for *H. longicornis* nymphs, carvacrol (LC_50_, 8.21 μg/cm^3^) exhibited the most effective acaricidal action, surpassing diethyltoluamide (LC_50_, 28.61 μg/cm^3^), followed by thymol (LC_50_, 10.37 μg/cm^3^) and permethrin (LC_50_, 18.24 μg/cm^3^). Carvacrol exhibited the most effective acaricidal activity against *H. longicornis* adults (LC_50_, 15.27 μg/cm^3^), surpassing diethyltoluamide (LC_50_, 48.58 μg/cm^3^), followed by thymol (LC_50_, 17.92 μg/cm^3^) and permethrin (LC_50_, 33.72 μg/cm^3^). The acaricidal efficacy of carvacrol and thymol, as indicated by the LC_50_ values, exhibited significant toxicity against *H. longicornis* larvae, nymphs, and adults. According to the LC_90_ values, carvacrol exhibited approximately 5.2-, 3.7-, and 3.3-fold greater efficacy than diethyltoluamide against *H. longicornis* larvae, nymphs, and adults, respectively. According to the LC_90_ values for *H. longicornis* larvae, carvacrol (LC_90_, 7.61 μg/cm^3^) exhibited the most effective acaricidal activity, surpassing diethyltoluamide (LC_90_, 39.57 μg/cm^3^), followed by thymol (LC_90_, 9.31 μg/cm^3^) and permethrin (LC_90_, 21.15 μg/cm^3^). According to the LC_90_ values against *H. longicornis* nymphs, carvacrol (LC_90_, 14.68 μg/cm^3^) exhibited the most effective acaricidal activity, surpassing diethyltoluamide (LC_90_, 54.29 μg/cm^3^), followed by thymol (LC_90_, 17.95 μg/cm^3^) and permethrin (LC_90_, 31.57 μg/cm^3^). Carvacrol exhibited the most effective acaricidal activity against *H. longicornis* adults (LC_90_, 24.17 μg/cm^3^), surpassing diethyltoluamide (LC_90_, 78.64 μg/cm^3^), followed by thymol (LC_90_, 28.14 μg/cm^3^) and permethrin (LC_90_, 62.87 μg/cm^3^).

The results of this study underscore the potent acaricidal efficacy of carvacrol and thymol against *H. longicornis* across different developmental stages. The immersion assay demonstrated that both compounds exhibited significantly lower LC_50_ and LC_90_ values compared to synthetic acaricides, diethyltoluamide and permethrin, indicating their potential as effective natural alternatives. Among the two, carvacrol exhibited superior acaricidal potency, with efficacy approximately 4.4-, 3.5-, and 3.2-fold greater than diethyltoluamide against larvae, nymphs, and adults, respectively. This trend was also evident in the LC_90_ values, where carvacrol outperformed diethyltoluamide by 5.2-, 3.7-, and 3.3-fold, further confirming its strong toxic effects on *H. longicornis*. These findings align with previous research suggesting that monoterpenoid phenols, particularly carvacrol and thymol, disrupt the nervous system of arthropods by modulating neuroreceptor functions and interfering with enzyme activity [8,11]. Carvacrol, a major constituent of *O. vulgare* oil, is known for its ability to induce oxidative stress and disrupt ion transport across cell membranes, leading to paralysis and mortality in the target organisms [9]. This mechanism likely contributes to its heightened acaricidal efficacy against *H. longicornis*. Interestingly, while thymol also demonstrated significant toxicity, it was consistently less effective than carvacrol. This difference in efficacy may be attributed to subtle structural variations between the two compounds that influence their interactions with mite neuroreceptors and metabolic pathways. Prior studies have suggested that while both thymol and carvacrol exert neurotoxic effects, carvacrol’s hydroxylation pattern enhances its binding affinity to insect and arachnid ion channels, resulting in stronger acaricidal activity [12,13]. A particularly important observation from this study is that carvacrol and thymol exhibited higher efficacy than permethrin, a widely used synthetic pyrethroid. Given the rising incidence of mite resistance to synthetic acaricides, these natural compounds present a promising alternative for mite control. Moreover, essential oil-derived acaricides are considered more environmentally friendly and pose a lower risk of toxicity to non-target organisms, making them attractive candidates for integrated pest management strategies [14,15].

### 2.5. AChE Activity of Carvacrol Against H. longicornis Nymphs

An in vitro study of AChE activity was conducted to test the inhibitory effects of carvacrol, the acaricidal chemical present in the essential oils of *O. vulgare* leaves, on *H. longicornis* nymphs. This study demonstrated that carvacrol at 30, 20, 10, 5, and 2.5 mg/mL produced AChE activity of 9.23, 21.47, 29.89, 32.87, and 41.13 nmol/min/mg protein, respectively. The acetone control group demonstrated an AChE activity of 90.82 nmol/min/mg protein. The AChE activity of the control group was established as the baseline (100%), whereas the AChE activity of the carvacrol-treated groups was represented as a percentage in relation to the control. Carvacrol diminished AChE activity to 14.6%, 34.1%, 47.4%, 52.1%, and 65.4% at 30, 20, 10, 5, and 2.5 mg/mL, respectively. This demonstrated that carvacrol inhibited AChE activity by 85.4–34.8% relative to the control group in a dose-dependent fashion. To further validate the AChE inhibitory efficacy of carvacrol in vivo, *H. longicornis* nymphs were subjected to its LC_50_. Following 24 h exposure period, the treated nymphs demonstrated an AChE activity of 68.25 nmol/min/mg protein, while the nymphs in the negative control exhibited an AChE activity of 92.14 nmol/min/mg protein. This indicates a decrease in the AChE activity to 74.1% of the control level, reflecting a statistically significant inhibition of 25.9%. These findings indicate that carvacrol significantly suppresses AChE activity in both in vitro and in vivo settings. The results indicate that the acaricidal action of carvacrol on *H. longicornis* nymphs may stem from its capacity to block AChE activity, thus interrupting vital physiological processes necessary for survival. The primary component of *Lippia gracilis* oil, carvacrol, has been found to be highly effective against *Rhipicephalus microplus* nymphs (LC_50_, 0.22 mg/mL) [16]. In a comparison with permethrin, carvacrol achieved a 100% mortality against *H. longicornis* larvae at 1–5% after 24 h, surpassing permethrin in terms of efficacy [9].

Essential oils and plant-derived chemicals exhibit multiple mechanisms for pest control and insecticidal activity, including neurotoxicity, disruption of growth regulation, and suppression of detoxifying enzymes [17]. AChE is an enzyme that hydrolyzes the neurotransmitter acetylcholine and plays a crucial role in the neurological system of pests and mites. Inhibition of AChE activity leads to the accumulation of acetylcholine, resulting in the paralysis and eventual mortality of the pests [18,19]. The inhibition of AChE activity constitutes a primary mechanism of action for organophosphate and carbamate insecticides, with numerous plant essential oils and plant-derived compounds also recognized for their capacity to inhibit AChE as a principal mechanism of their insecticidal and acaricidal effects [18,19]. This study confirmed through in vitro and in vivo AChE activity assays that carvacrol significantly inhibits AChE activity in *H. longicornis* nymphs, indicating that this inhibitory activity is a primary mechanism of its acaricidal effect on the nymphs (Table 5).

The effectiveness of natural acaricides is underscored by the strong action of carvacrol against *H. longicornis*, a principal vector of SFTS, demonstrating considerable toxicity at all developmental stages. This corresponds with research on *Rhipicephalus microplus*, wherein carvacrol and thymol exhibited synergistic effects when used in conjunction with cypermethrin, hence augmenting their acaricidal efficacy against resistant tick populations [20]. The lack of cross-resistance between these natural acaricides and synthetic acaricides indicates their potential efficacy against ticks resistant to standard treatments. Carvacrol functions by reducing the AChE activity and suppressing the detoxifying enzymes, potentially aiding in the mitigation of resistance processes linked to synthetic chemicals. The effectiveness of this chemical highlights the promise of botanical substances in tick management, providing safer options compared to synthetic acaricides. Carvacrol and cinnamaldehyde have the capability to mitigate resistance and diminish environmental toxicity, rendering them appealing options for integrated pest management systems.

### 2.6. Detoxifying Enzyme Activity

**Figure 1:**
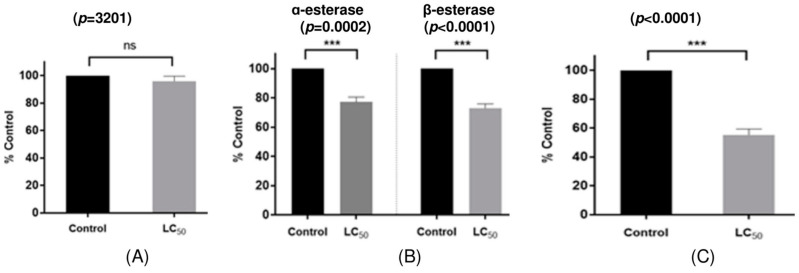
In vivo enzyme activity of GST, α-esterase, β-esterase, and P450s activities in *H. longicornis* nymphs treated with carvacrol at LC50. Error bars represent the standard error of the mean. Asterisks indicate significant differences when compared to respective control (unpaired *t* test, *p* < 0.001); ns, not significant. (**A**) Glutathion S-transferase, (**B**) General esterase, (**C**) Cytochrome P450 monooxygenases.

This study investigates the alterations in detoxifying enzyme activity in *H. longicornis* nymphs subjected to carvacrol, specifically assessing the activities of glutathione *S*-transferase (GST), α-/β-esterase, and cytochrome P450 monooxygenases (P450s) in *H. longicornis* nymphs exposed to LC_50_ concentration of carvacrol (Table 6). The control group subjected to 50% ethanol demonstrated α-esterase, β-esterase, and P450s activities of 730.2, 595.6, and 2.83 nmol/min/mg protein, respectively, whereas *H. longicornis* nymphs exposed to the LC_50_ concentration of carvacrol displayed enzyme activities of 549.4, 425.4, and 1.49 nmol/min/mg protein, respectively. No statistically significant difference in GST activity (*p* < 0.05) was detected between the control group (1.60 µmol/min/mg protein) and the carvacrol-exposed *H. longicornis* nymphs (1.54 µmol/min/mg protein) (Table 6 and Figure 1). With the enzyme activity of the control group established at 100%, the activities of α-esterase, β-esterase, and P450s in *H. longicornis* nymphs exposed to carvacrol were quantified as percentages of control, yielding 75.2%, 71.4%, and 52.6%, respectively. This demonstrated that the enzyme activities were decreased by 24.8%, 28.6%, and 47.4%, respectively, in comparison to the control group (Figure 1).

Detoxifying enzymes in pests, including GST, P450s, and α-/β-esterase, are crucial for sustaining physiological activities by neutralizing chemicals such as pesticides [21]. GST augments the defensive powers of insects and mites by stimulating the development of more detoxifying enzymes, therefore facilitating cross-resistance to pesticides. P450s are hydrophobic enzymes that participate in the metabolism of several endogenous and exogenous chemicals, as well as in the oxidation of insecticides and acaricides [22]. β-esterase in insects and mites is crucial for the detoxification of exogenous compounds and the development of resistance, while α-/β-esterase can modify the vulnerability of pests and mites to insecticides by detoxifying them post-binding to specific insecticides [23]. This study found that the general esterase and P450s activities in *H. longicornis* nymphs exposed to carvacrol were considerably diminished compared to the control group, although GST activity exhibited no significant variation from the control group. Mites subjected to acaricidal substances may generate increased levels of detoxifying enzymes to mitigate the effects, resulting in a notable rise in detoxifying enzyme activity during the initial phases. Nonetheless, the prolonged impact of acaricidal agents may lead to a steady decline in enzyme function. Huang et al. [24] documented that in their investigation of the alterations in GST, P450s, α-esterase, and β-esterase activities in *Aedes albopictus* subjected to linalool and carvacrol, GST activity in *A. albopictus* exposed to linalool initially rose but significantly declined after 12 h, whereas *A. albopictus* exposed to carvacrol exhibited a notable reduction in the activities of all detoxification enzymes. Kumrungsee et al. [21] indicated that thymol and 1,8-cineole demonstrated significant insecticidal efficacy against *Plutella xylostella* nymphs, while concurrently enhancing the activities of GST, α-/β-esterase, and carboxylesterase, implying that this elevation in detoxifying enzyme activity may facilitate the emergence of resistance in *P. xylostella*. This study indicates that carvacrol, which exhibited inhibitory effects on P450s, and α-/β-esterase in *Tetranychus urticae* nymphs, is unlikely to contribute to resistance development in *H. longicornis* nymphs and is believed to be effectively applicable to resistant individuals exhibiting elevated detoxifying enzyme activity induced by insecticides.

Carvacrol has been evaluated for its toxicity in humans, livestock, and non-target organisms, showing a generally low acute toxicity but requiring further research on long-term effects. Studies indicate that it is safe as a feed additive for livestock at moderate doses, but may cause oxidative stress at higher concentrations [25]. It exhibits minimal toxicity to beneficial insects like dung beetles and earthworms, making it a potential eco-friendly pesticide alternative [26,27]. In human cell studies, carvacrol showed lower cytotoxicity than synthetic pesticides but may induce oxidative stress with prolonged exposure [28]. While promising, further studies are needed to ensure its environmental and health safety.

## 3. Materials and Methods

### 3.1. Chemicals and Sample Preparation

α-Terpinene (EC 202-795-1), cymene (EC 202-796-7), γ-terpinene (EC 202-794-6), borneol (CAS 464-45-9), linalool (EC 201-134-4), thymol (CAS 89-83-8), thymoquinone (CAS 490-91-5), caryophyllene (EC 214-519-7), permethrin (CAS 1794760-19-2), diethyltoluamide (EC 205-149-7), acetylthiocholine iodide (EC 217-474-1), calcium sulfate hemihydrate (EC 231-900-3), 1-chloro-2,4-dinitrobenzene (EC 202-551-4), 5,5′-dithiobis-(2-nitro benzoic acid) (DTNB) (CAS 69-78-3), Fast blue BB solution (CAS 5486-84-0), (-)-globulol (EC 207-696-7), glutathione (CAS 70-18-8), 3,3,5,5-tetramethylbenzidine (EC 259-364-6), and Tween 80 (CAS 9005-65-6) were procured from Sigma−Aldrich (St. Louis, MO, USA). The carvacrol used in all experiments was isolated. *O. vulgare* leaves (10 kg) obtained from markets in Seoul and Suwon, Republic of Korea, were cultivated in Germany (Bavaria: 48.1351° N, 11.5820° E), Albania (Berat: 40.7083° N, 19.9440° E), and Iran (Lorestan Province: 33.4877° N, 48.3558° E), respectively. Their corresponding voucher numbers are as follows: German *O. vulgare* (Voucher No. HS8237), Albanian *O. vulgare* (Voucher No. HS8238), and Iranian *O. vulgare* (Voucher No. HS8239) in Herbarium HS biotech, Korea. They were cleansed three times, desiccated, and then ground into powder. The extraction of essential oil from powdered *O. vulgare* leaves was achieved using steam distillation with a Clevenger apparatus. A substantial quantity of dried *O. vulgare* powder was placed in the distillation flask. This flask was connected to a steam generator that produced steam. As the steam interacted with the power, it extracted the essential oils from the plant cells, conveying them upward into the condenser. The condenser cooled the steam, causing it to revert to liquid form. The liquid, consisting of a combination of water and essential oil, then proceeded to the separation chamber of the Clevenger apparatus. The essential oil separated from the water due to differences in density, allowing for the collection of the oil in a separate layer.

### 3.2. GC/MS Analysis

The essential oil from *O. vulgare* leaves cultivated in Germany was examined using GC/MS (HP 6890 GC and 5973 Mass, Agilent Technologies, Santa Clara, CA, USA) equipped with a 0.25 mm DB-5 and a fused-silica capillary column (30 m × 0.25 mm). At a rate of 2.0 °C/min, the initial oven temperature of 62 °C was raised to 212 °C and maintained for 8.9 min. After that, the oven temperature was raised to 240 ± 1.5 °C for 15 min at a rate of 2.1 °C/min. The temperatures of the ion source and injector were set at 211 ± 2 °C. The analyzer was configured to scan between 50 and 560 amu, and spectra were obtained at an ionization energy of 70 eV. Using retention duration and retention index as references, the components of *O. vulgare* oil were identified by comparing them to a mass spectrum library.

### 3.3. Isolation and Identification

**Figure 2:**
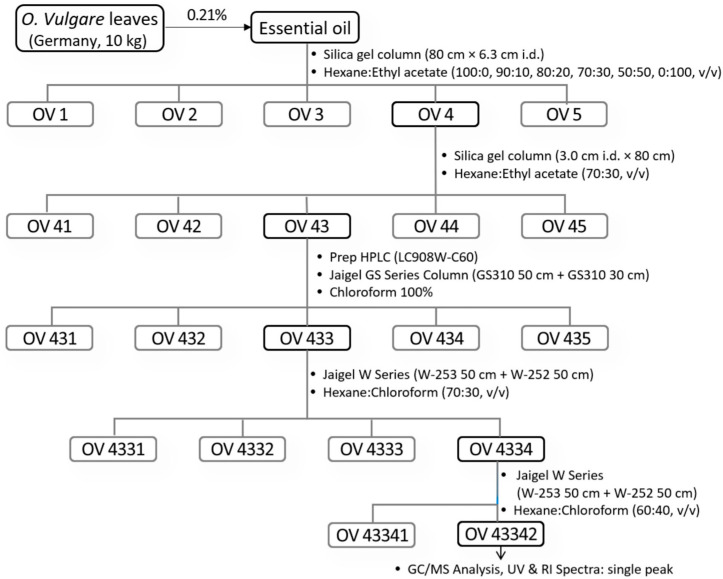
Isolation of carvacrol.

German *O. vulgare* oil (5 g) was dissolved in ethyl acetate and fractionated using a silica gel column (80 cm × 6.3 cm i.d.) packed with 230-mesh silica gel (Figure 2). A hexane:ethyl acetate gradient solvent system (100:0, 90:10, 80:20, 70:30, 50:50, and 0:100, *v*/*v*) was used at a flow rate of 2.5 mL/min to separate the oil into five fractions (OV1–OV5). Among these fractions, OV4 was identified as the active fraction based on its biological activity. OV4 was further purified using another silica gel column (3.0 cm i.d. × 80 cm) with a hexane:ethyl acetate mixture (70:30, *v*/*v*) as the mobile phase. Fractions with similar patterns, confirmed by TLC, were combined into sub-fractions labeled OV41 to OV45. OV43 was identified as the most active component. To analyze and purify OV43, its peak absorption wavelength was determined using a UV spectrophotometer. Recycling prep HPLC (Model: LC908W-C60) equipped with a Jaigel GS Series Column (GS310 50 cm + GS310 30 cm, Japan Analytical Industry Co., Ltd., Tokyo, Japan) was employed for separation. The mobile phase used was pure chloroform at a flow rate of 4.9 mL/min, and detection was performed at 290 nm. OV43 was further divided into five layers, among which OV433 showed the highest biological activity and was selected for bioassay testing. Further purification of OV433 was carried out using the Jaigel W Series Column (W-253 50 cm + W-252 50 cm) under optimized conditions: a flow rate of 4.8 mL/min, detection wavelength of 290 nm, and a hexane:chloroform mobile phase (70:30, *v*/*v*). Four fractions were obtained, and OV4334 exhibited the highest activity. By adjusting the mobile phase to hexane:chloroform (60:40, *v*/*v*) and performing three recycling cycles, a single active layer was isolated. TLC and GC/MS analysis confirmed that OV43342 displayed a single peak in UV and RI spectra, identifying it as the final active component. Spectroscopic methods, encompassing EI-MS, FT-IR, ^1^H NMR, DEPT NMR, ^13^C NMR, ^1^H-^1^H COSY, and ^1^H-^13^C HMQC NMR, were employed to elucidate the structures of OV43342. A mass spectrometer operating in electron ionization mode with electrospray was utilized to obtain the spectra. The mass spectrometry characteristics included a capillary voltage of 2.90 kV, negative ionization mode, a mass range of 10–300 eV, a column temperature that steadily increased from 50 °C to 202 °C, and a source temperature of 239 °C. The analysis employed FT-IR within the frequency range of 1000 to 4000 cm^−1^, utilizing a dedicated FT-IR. NMR measurements of ^13^C and ^1^H were conducted utilizing a JNM EX-600 (JEOL Co., Tokyo, Japan) with a magnetic field strength of 600 MHz and using deuterated chloroform (CDCl_3_) as the solvent.

### 3.4. Target Arachnids

*H. longicornis* larvae, nymphs, and adults were collected from cattle farms using the dragging method in Seogwipo (33.2538° N, 126.5616° E) and Jeonju (35.8250° N, 127.1486° E), Korea, from July to November. The larvae, nymphs, and adults were collected by dragging a white cotton cloth (100 × 90 cm) across the grass at ground level. The *H. longicornis* larvae, nymphs, and adults were chosen because of their lack of exposure to acaricides. The larvae, nymphs, and adults were morphologically identified following the approach presented by Baker and Walker [1]. The storage container was filled with a mixture of charcoal powder, CaSO_4_, and water to a depth of 6 cm and allowed to cure at room temperature for 24 h. The larvae, nymphs, and adults were preserved in a container with a top that permitted air circulation. To maintain a uniform moisture level, a suitable amount of water was provided to the gypsum. The larvae, nymphs, and adults were transferred to the incubator 48 h before the study began. They were subsequently cultured in a regulated environment at 26 ± 2 °C and 72 ± 2% relative humidity (RH).

### 3.5. Acaricidal Toxicity Bioassays and Data Analysis

The immersion bioassay was performed on *H. longicornis* larvae, nymphs, and adults, utilizing the approach outlined by Baker and Walker [1]. Thirty nymphs and larvae were submerged in a 10 mL sample solution with concentrations varying from 2 to 60 μg/cm^3^ for a duration of 5 min. Subsequent to immersion, the nymphs and larvae were placed in a Petri dish (1.5 cm × 5.5 cm) lined with filter paper for 10 min, during which the solvent was allowed to evaporate. Petri dishes with treated nymphs and larvae were incubated at 28 °C and 85–90% RH for 24 h. Adult immersion tests were conducted on unfed *H. longicornis* adults. In the immersion assay, thirty unfed *H. longicornis* adults were submerged for 5 min in test solutions with concentrations varying from 2 to 60 μg/cm^3^. Following immersion, *H. longicornis* adults were desiccated on Petri dishes lined with filter paper for 10 min, after which the dishes were incubated at 28 °C with 85–90% RH. After 24 h, the deceased *H. longicornis* specimens were enumerated using microscopic analysis. The negative control was immersed in a solution comprising 1% DMSO and 50% ethanol, while diethyltoluamide and permethrin served as the positive control. The acaricidal effectiveness of the substances was assessed against *H. longicornis* nymphs using the nymphal packet method [29]. The diluted sample (500 μL) at various concentrations was deposited onto filter paper, and the solvent was evaporated for 10 min in a fume hood. The filter paper was then folded in half, inoculated with 30 *H. longicornis* nymphs, and fastened with a bulldog clip. The sealed filter paper was kept at 26 ± 2 °C and 80% RH. The results were confirmed after 24 h using a stereoscopic microscope (SZX7, Olympus, Tokyo, Japan), and the acaricidal efficacy was assessed using Abbott’s formula [30].

### 3.6. In Vitro and In Vivo AChE Activity

The AChE activity was assessed using a spectrophotometric assay [17]. The *H. longicornis* nymphs were exposed to the LC_50_ of the sample, as previously established in the nymphal package assay. In vitro and in vivo AChE activity, only the individuals that passed the test were used in this experiment. Furthermore, the nymphs were promptly exposed to homogenization using a homogenizer. Subsequently, the homogenate underwent a 3 min homogenization at 4 °C, employing 1.5 mL of a buffer consisting of 0.1 M Na_3_PO_4_ and 0.1% Triton X-100. The sample was subsequently centrifuged at 10,000 rpm for an additional 30 min at 4 °C. Thereafter, the supernatant was gathered and preserved at 4 °C for use as an enzyme. The enzyme obtained from *H. longicornis* nymphs treated with 50% ethanol was utilized as a negative control. The protein concentration was quantified utilizing the Bradford protein assay [31]. AChE activity was assessed by combining 2.8 mL of 0.1 M phosphate buffer (pH 7.5), 100 μL of 0.01 M DTNB, 50 μL of enzyme, and 20 μL of sample (30, 20, 10, 5, and 2.5 mg/mL) in a cuvette. The enzymatic reaction commenced with the addition of 0.075 M acetylthiocholine iodide (50 μL). The hydrolysis of the substrate was evaluated by comparing the hydrolysis rate in a blank sample devoid of acetylthiocholine iodide to that in a blank sample containing 50% ethanol instead of carvacrol. The 2-nitro-5-thiobenzoate anion, generated from the interaction between thiocholine and DTNB, was quantified at 412 nm for 10 min utilizing a UV-1800 spectrophotometer (Shimadzu, Japan). The absorption kinetics were assessed within a linear range and subsequently converted to micromoles per minute per milligram of protein based on the molar extinction coefficient (*ε* = 1.36 × 10^4^ L/mol/cm).

### 3.7. Detoxifying Enzyme Activity Method

The activities of GST, α-/β-esterase, and P450s were evaluated using established methodologies [32]. The enzyme preparation involved homogenizing thirty live ticks in 0.1 M phosphate buffer (pH 7.5) at 0 °C, followed by centrifugation at 10,000 rpm for 30 min at 4 °C. The supernatant was used as the enzyme source, and protein concentration was determined using the Bradford protein assay [31], as described previously for AChE activity. GST activity: Utilized 1-chloro-2,4-dinitrobenzene as the substrate. The reaction mixture consisted of 2.5 mL of 0.1 M phosphate buffer (pH 7.5), 50 μL of enzyme, 100 μL of 1-chloro-2,4-dinitrobenzene, and 300 μL of reduced glutathione. Measurements were taken at 340 nm at 60 s intervals over 10 min. GST activity was quantified as μmol/mg protein/min (ε = 0.0096 μM^−1^cm^−1^). α-/β-Esterase activities: Employed α-naphthyl acetate and β-naphthyl acetate as substrates [23], following the method of Kao et al. [33]. The reaction involved combining 100 μL of enzyme with 2.5 mL of α-naphthyl acetate/β-naphthyl acetate in a cuvette and incubating at 35 °C. After 20 min, Fast Blue BB solution was added, and absorbance was recorded at 605/555 nm. Activity was quantified as nmol/mg protein/min using the standard curve of α-naphthol/β-naphthol. P450s activity: Evaluated using the methodology of William et al. [34]. The reaction consisted of 0.0625 M potassium phosphate buffer (0.8 mL), 3,3,5,5-tetramethylbenzidine (2.25 mL), 3% hydrogen peroxide, and enzyme (100 μL), incubated at 25 °C for 30 min. Measurements were taken at 650 nm. P450s activity was quantified as nmol/mg protein/min using the standard curve of cytochrome C.

### 3.8. Statistical Analysis

To determine the acaricidal efficacy of carvacrol and other components against *H. longicornis*, statistical analyses were conducted using various methods. The 50% and 90% lethal dose (LC_50_ and LC_90_) values, along with the slopes of the regression lines, were calculated using SPSS Statistical Package (version 12). All results were expressed as mean ± standard error of the mean ± SEM using GraphPad Prism (version 10 Academic, San Diego, CA, USA). Significant differences between treatment groups were assessed using one-way analysis of variance (ANOVA) followed by Tukey’s post hoc test. For comparisons between treatment and negative control groups regarding enzyme activity, Student’s unpaired *t*-test was employed. To ensure the validity of parametric tests, the normality of the data distribution was verified. For small sample sizes (n < 50), the Shapiro–Wilk test was used, while the Kolmogorov–Smirnov test was applied for larger datasets (n ≥ 50). Additionally, Q-Q plot analysis was performed to visually assess normality. Only datasets meeting the assumption of normality were analyzed using parametric methods; otherwise, appropriate non-parametric tests were applied. A probit regression analysis was conducted to evaluate the dose–response relationship of acaricidal activity. The coefficient of determination (R^2^) was calculated to assess the goodness-of-fit of the model, indicating the proportion of variance in mortality rates explained by the regression. This comprehensive statistical approach ensured robust and reliable conclusions regarding the efficacy of carvacrol as a natural acaricide against *H. longicornis*.

## 4. Conclusions

This study emphasizes the acaricidal efficacy of carvacrol, a principal phenolic monoterpenoid derived from *O. vulgare* oil, against *H. longicornis*. Carvacrol exhibited notable acaricidal efficacy at all developmental stages, surpassing synthetic acaricides. The efficacy is ascribed to its capacity to inhibit AChE activity, alter neuronal communication, and suppress detoxifying enzymes such P450s and α-/β-esterase, resulting in paralysis and mortality in *H. longicornis*. Chemical study verified that carvacrol is the principal active constituent in *O. vulgare* oil, with the highest concentration in German oil. Despite the identification of other active chemicals such as thymol and thymoquinone, carvacrol’s prevalence and efficacy designated it as the principal contributor to acaricidal actions. In comparison to synthetic acaricides, carvacrol shown enhanced toxicity profiles, suggesting its viability as an effective natural option for controlling *H. longicornis*. These findings indicate that carvacrol could function as an environmentally sustainable method for controlling *H. longicornis* populations while mitigating concerns related to resistance and ecological toxicity. Future research should study on enhancing carvacrol-based formulations for practical uses and investigating synergistic interactions with other natural chemicals.

## Figures and Tables

**Table 1:** Acaricidal activities of the essential oils of *O. vulgare* leaves cultivated in Germany, Albania, and Iran and commercial acaricide against *H. longicornis*, using immersion assay ^a^.

Essential Oils	Life Stage	LC_50_ (95% CL) (μg/cm^3^)	LC_90_ (95% CL) (μg/cm^3^)	Slope ± SE	R^2^ Value	χ^2^ Value (df, *p*)	*p* Value ^b^	RT ^c^
Germany oil	Larvae	3.53 (2.89–4.41)	7.15 (5.11–11.18)	4.98 ± 0.52	0.915	0.59 (4, 0.78)	-	4.4
	Nymph	12.32 (9.87–15.15)	24.14 (20.15–31.01)	3.87 ± 0.51	0.942	1.02 (3, 0.61)	-	2.3
	Adult	18.14 (15.44–23.01)	40.74 (35.29–49.15)	3.19 ± 0.44	0.953	1.54 (3, 0.49)	-	2.7
Albania oil	Larvae	4.49 (3.67–5.38)	10.14 (8.14–14.95)	4.21 ± 0.68	0.934	0.69 (4, 0.71)	0.032	3.4
	Nymph	13.86 (11.95–16.89)	32.18 (27.15–41.19)	4.01 ± 0.59	0.961	1.56 (3, 0.62)	0.045	2.1
	Adult	21.02 (18.06–25.34)	44.16 (39.18–55.57)	4.99 ± 0.51	0.958	2.41 (3, 0.69)	0.048	2.3
Iran oil	Larvae	5.27 (4.58–6.11)	14.21 (9.68–20.26)	5.19 ± 0.71	0.917	0.76 (4, 0.68)	0.024	2.9
	Nymph	15.02 (12.27–19.27)	36.54 (30.12–43.28)	5.12 ± 0.63	0.924	1.14 (3, 0.59)	0.029	1.9
	Adult	25.29 (19.64–31.24)	48.19 (40.16–59.28)	3.97 ± 0.59	0.964	1.97 (3, 0.49)	0.035	2.0
Diethyltoluamide ^d^	Larvae	15.34 (13.42–17.41)	39.39 (37.25–42.91)	4.14 ± 0.63	0.894	2.91 (3, 0.54)	<0.001	1.0
	Nymph	28.61 (26.27–31.54)	54.29 (50.71–57.89)	3.91 ± 0.61	0.872	2.81 (4, 0.59)	<0.001	1.0
	Adult	49.21 (46.41–52.15)	72.46 (65.09–83.04)	5.01 ± 0.73	0.863	5.29 (4, 0.41)	<0.001	1.0

^a^ Exposed for 24 h. ^b^ The German oil was the most effective at all stages, and the difference was statistically significant (*p* < 0.05). ^c^ RT, Relative toxicity = LC_50_ value of diethyltoluamide/LC_50_ value of each compound. ^d^ Commercial acaricide as positive control.

**Table 2:** Analysis of the constituents of the essential oils of *O. vulgare* leaves cultivated in Germany, Albania, and Iran identified by GC/MS.

Chemical	Classification	Retention Time (min)	Retention Index	Relative (%)					
				Germany		Albania		Iran	
α-Terpinene	Monoterpene Hydrocarbons	6.28	1018	0.30	0.3%	0.46	0.5%	2.41	2.5%
Cymene	Monoterpene Hydrocarbons	6.42	1026	4.94	5.0%	5.59	5.6%	6.34	6.5%
γ-Terpinene	Monoterpene Hydrocarbons	7.06	1062	2.77	2.8%	3.33	3.4%	5.36	5.5%
Borneol	Monoterpene Alcohols	10.03	1108	0.49	0.5%				
Linalool	Monoterpene Alcohols	10.14	1115	2.36	2.4%	2.98	3.0%	4.98	5.1%
Thymol	Phenols	11.38	1302	4.72	4.8%	5.74	5.8%	8.68	8.9%
Carvacrol	Phenols	11.66	1310	83.38	83.9%	79.66	80.1%	63.86	65.6%
Thymoquinone	Quinones	12.15	1359					0.23	0.2%
Caryophyllene	Sesquiterpenes	13.09	1420	0.41	0.4%	1.75	1.8%	5.52	5.7%

**Table 3:** Acaricidal activities of the constituents of *O. vulgare* oil and commercial acaricides against *H. longicornis* nymphs, using immersion assay ^a^.

Compounds	LC_50_ (95% CI) ^b^	LC_90_ (95% CI) ^b^	Slop ± SE	*p* Value ^c^	χ^2^ (df, *p*)	RT_50_ ^d^
α-Terpinene	−	−	−	ns ^f^	−	−
Cymene	−	−	−	ns	−	−
γ-Terpinene	−	−	−	ns	−	−
Borneol	−	−	−	ns	−	−
Linalool	−	−	−	ns	−	−
Thymol	10.37 (8.59–12.24)	17.95 (14.49–23.65)	5.19 ± 0.68	<0.05	1.22 (4, 0.76)	2.8
Carvacrol	8.21 (7.19–9.94)	14.68 (11.87–20.56)	5.31 ± 0.79	<0.01	1.09 (4, 0.74)	3.5
Thymoquinone	4.98 (3.56–6.24)	9.67 (7.56–14.21)	4.68 ± 0.79	<0.01	1.68 (3, 0.57)	5.8
Caryophyllene	−	−	−	ns	−	−
Permethrin ^e^	18.24 (15.29–24.67)	31.57 (25.64–43.51)	4.17 ± 0.72	<0.01	2.17 (4, 0.81)	1.6
Diethyltoluamide ^e^	28.61 (26.27–31.54)	54.29 (50.71–57.89)	3.91 ± 0.61	<0.01	2.81 (4, 0.59)	1.0

^a^ LC_50_ is the average of four determinations, with 30 mites per replication. ^b^ 95% CI: Compounds activity is considered significantly different when the 95% CI fail to overlap. ^c^ Statistical significance of the difference between the treatment and the negative control medians. ^d^ RT_50_, relative toxicity = LC_50_ value of diethyltoluamide/LC_50_ value of each compound. ^e^ Commercial acaricides as positive control. ^f^ No statistically significant difference.

**Table 4:** Acaricidal activities of carvacrol and thymol derived from *O. vulgare* oil and commercial acaricides against *H. longicornis*, using immersion assay ^a^.

Compounds		LC_50_ (95% CI) ^b^ (μg/cm^3^)	LC_90_ (95% CI) ^b^ (μg/cm^3^)	Slop ± SE	χ^2^ (df, *p*)	RT_50_ ^c^
Thymol	Larvae	5.26 * (4.14–6.85)	9.31 (7.19–11.63)	3.98 ± 0.52	4.36 (4, 0.52)	2.9
	Nymphs	10.37 * (8.59–12.24)	17.95 (14.49–23.65)	5.19 ± 0.68	1.22 (4, 0.76)	2.8
	Adults	17.92 * (13.95–22.64)	28.14 (24.95–32.63)	3.65 ± 0.38	2.56 (3, 0.52)	2.7
Carvacrol	Larvae	3.47 * (2.14–4.92)	7.61 (5.25–9.87)	2.67 ± 0.35	4.31 (3, 0.31)	4.4
	Nymphs	8.21 * (7.19–9.94)	14.68 (11.87–20.56)	5.31 ± 0.79	1.089 (4, 0.74)	3.5
	Adults	15.27 * (11.19–19.98)	24.17 (21.27–28.66)	4.28 ± 0.62	2.96 (4, 0.56)	3.2
Permethrin ^d^	Larvae	10.24 * (8.13–14.47)	21.15 (16.38–30.29)	4.96 ± 0.81	3.14 (3, 0.59)	1.5
	Nymphs	18.24 * (15.29–24.67)	31.57 (25.64–43.51)	4.17 ± 0.72	2.17 (4, 0.81)	1.6
	Adults	33.72 (23.64–45.54)	62.87 (49.28–79.64)	5.68 ± 0.76	3.59 (4, 0.77)	1.4
Diethyltoluamide ^d^	Larvae	15.31 * (12.21–19.32)	39.57 (35.08–45.22)	3.69 ± 0.53	3.21 (3, 0.56)	1.0
	Nymphs	28.61 * (26.27–31.54)	54.29 (50.71–57.89)	3.91 ± 0.61	2.81 (4, 0.59)	1.0
	Adults	48.58 * (43.25–55.59)	78.64 (62.52–89.43)	4.65 ± 0.74	5.67 (4, 0.48)	1.0

^a^ LC_50_ is the average of five determinations, with 30 mites per replication. ^b^ 95% CI: Compounds activity is considered significantly different when the 95% CI fail to overlap. ^c^ RT_50_, Relative toxicity = LC_50_ value of diethyltoluamide/LC_50_ value of each compound. ^d^ Commercial acaricides as positive control. * Significantly different from the control (Tukey HSD test, *p* < 0.05).

**Table 5:** In vitro and in vivo AChE activity of carvacrol against *H. longicornis* nymphs.

Sample		Conc. (mg/mL)	AChE Activity (nmol/min/mg Protein ± SE)	Inhibition (%)	*p*-Value
Carvacrol	In vitro	30	9.23 ± 0.45	85.4	<0.05 ^a^
		20	21.47 ± 0.86	65.9	<0.05
		10	29.89 ± 0.82	52.6	<0.05
		5.0	32.87 ± 0.58	47.9	<0.05
		2.5	41.13 ± 0.85	34.8	<0.05
	In vivo	LC_50_ (8.21)	68.25 ± 1.32	25.9	<0.01 ^b^

^a^ Significantly different from the control (Tukey HSD test). ^b^ Significantly different from the control (unpaired *t* test).

**Table 6:** GST, general esterase, and P450s activities in *H. longicornis* nymphs exposed to LC_50_ of carvacrol.

Treatment (mg/mL)	GST (µmol/min/mg Protein ± SE)	α-Esterase	β-Esterase	P450s (nmol/min/mg Protein ± SE)
		(nmol/min/mg Protein ± SE)		
Control	1.60 ± 0.07	730.2 ± 22.1	595.6 ± 18.6	2.83 ± 0.06
LC_50_ (8.21)	1.54 ± 0.06	549.4 ± 26.7 *	425.4 ± 21.3 *	1.49 ± 0.08 *

* Significantly different from the control (unpaired *t* test, *p* < 0.01).

## Data Availability

The data that support the findings of this study are present in the main manuscript. Additional data related to this paper may be obtained from the corresponding author on reasonable request.
